# Antityphoid Vaccination

**Published:** 1916-04

**Authors:** 


**Antityphoid Vaccination.** Leo Bernard (quoted in Med. World, Jan.) from a study of 800 cases of typhoid, states that the mortality was 5½% for the vaccinated and 17% for unvaccinated. He believes that the former rate would have been still lower if all had received a triple injection.

